# Fault Diagnostics for Turbo-Shaft Engine Sensors Based on a Simplified On-Board Model

**DOI:** 10.3390/s120811061

**Published:** 2012-08-09

**Authors:** Feng Lu, Jinquan Huang, Yaodong Xing

**Affiliations:** College of Energy and Power Engineering, Nanjing University of Aeronautics and Astronautics, Nanjing 210016, China; E-Mails: jhuang@nuaa.edu.cn (J.H.); huhotxudong@163.com (Y.X.)

**Keywords:** turbo-shaft engine, fault diagnosis, gas turbine sensor, simplified on-board model, dynamic parameters

## Abstract

Combining a simplified on-board turbo-shaft model with sensor fault diagnostic logic, a model-based sensor fault diagnosis method is proposed. The existing fault diagnosis method for turbo-shaft engine key sensors is mainly based on a double redundancies technique, and this can't be satisfied in some occasions as lack of judgment. The simplified on-board model provides the analytical third channel against which the dual channel measurements are compared, while the hardware redundancy will increase the structure complexity and weight. The simplified turbo-shaft model contains the gas generator model and the power turbine model with loads, this is built up via dynamic parameters method. Sensor fault detection, diagnosis (FDD) logic is designed, and two types of sensor failures, such as the step faults and the drift faults, are simulated. When the discrepancy among the triplex channels exceeds a tolerance level, the fault diagnosis logic determines the cause of the difference. Through this approach, the sensor fault diagnosis system achieves the objectives of anomaly detection, sensor fault diagnosis and redundancy recovery. Finally, experiments on this method are carried out on a turbo-shaft engine, and two types of faults under different channel combinations are presented. The experimental results show that the proposed method for sensor fault diagnostics is efficient.

## 1. Introduction

With ever-increasing demands being placed on modern aero-engine control systems, the number of control variables and sensors is increasing [1]. The fault-tolerant control and health management of aero-engines depend on accurate and reliable sensor readings, while most sensors work in the harsh environment of high temperature and strong vibration [2,3]. Most aircraft engines have been enhanced by equipping the engines with a dual channel full authority digital electronic control (FADEC) system [4,5]. A single channel failure is accommodated in FADEC by utilizing the measurement on the other channel, but how to recognize the failed channel can't be realized. Therefore, sensor fault diagnosis and isolation (FDI) plays a key role in enhancing safety, reliability and reducing the operating cost of aircraft propulsion systems.

Wallhagen and Arpasi proposed using analytical redundancy sensor technology to improve the reliability of the engine control system in 1974 [6]. The fault indication and correction action (FICA) system was developed by Spang and Corley, and is widely available [7]. The FICA system had been applied in the FADEC plan of T700, JTDE and F404, but it cannot detect soft failures. A generalized likelihood ratio approach for detection and estimation of jumps in a linear system is proposed by Willsky [8]. In order to detect and isolate the control system, sensor and actuator faults, and provide analytical redundancy, NASA developed an analytical redundancy design for engine reliability improvement [9,10]. The fault indices are mainly the thresholds of amplitude and slope, and built-in-test (BIT) technology is also used for sensor fault diagnosis in engineering.

The sensor fault diagnosis methods are generally divided into three categories: mathematical models-based, knowledge-based and signal processing-based [11–15]. Randal presented neural networks and multiple failures assumption methods for failure diagnosis of sensors and actuators [16]. Ogaji reported multiple-sensor fault-diagnoses for a 2-shaft stationary gas-turbine [17]. Zhernakov described diagnostics and checking of gas-turbine engines parameters with hybrid expert systems [18]. Aretakis discussed identification of sensor faults on turbofan engines using pattern recognition techniques [19]. Bettocchi presented a method based on analytical redundancy techniques for sensor fault detection in gas turbines. According to this method I/O linear models are used for residuals generation permitting the identification of possible sensor faults. Merrill used a bank of Kalman filters for detection, isolation, and accommodation of sensor failures [20,21]. Kobayashi developed a fault detection and isolation (FDI) system that utilizes a bank of Kalman filters for aircraft engine sensor and actuator FDI in conjunction with the detection of component faults [22]. A baseline system and an enhanced system utilizing dual-channel sensor measurements for aircraft engine on-line diagnostics are developed by Kobayashi in 2008 [23–25]. The algorithm, support vector regression trained by self-tuning particle swarm optimization (SPSO-SVR), was proposed and used to diagnose sensor faults in 2009 [26].

The model-based approach needs a pre-established engine model to acquire the analytic third channel, so the model is built up based on aircraft engine component characteristics and pneumatic thermodynamic equations. Compared to the methods of knowledge-based and signal processing-based, it avoids the difficult problems of knowledge base maintenance and high robustness requirements of measurement data, and can be used to diagnose new sensor failures with no need of history experience or *a priori* knowledge [27,28]. Kalman filter is one of the most typical model-based methods for sensor diagnostics on-line. However, the Kalman gain matrix calculation is iterative and requires higher performance of on-board hardware, therefore it is limited to on-line application on the ground. To overcome such problems, a simple and efficient approach should be adopted.

This article aims to apply the simplified on-board model as an analytical third channel for sensor fault diagnosis of turbo-shaft engines. This system is referred to as the sensor FDD system, and its structure is composed of the simplified on-board turbo-shaft engine and fault detection and diagnosis logic. Considering the measurement noises and modeling errors, sensor dual channel threshold and analytic thresholds are designed that are used for the logic. When the difference among the triplex channels violates a tolerance level, the logic determines the cause of the difference. Due to its simplicity, the sensor fault diagnosis system can be executed with the computing power currently available on line.

This paper is organized as follows: in Section 2, the simplified on-board turbo-shaft model for sensor fault diagnosis, which contains gas generator model and power turbine model with loads, is described. In Section 3, the pre-processes for measurements are presented, sensor fault diagnostic thresholds are calculated, and diagnostic logic is designed based on the simplified model. Experiments are carried out in Section 4, and the results show that compared to the SPSO-SVR method, the one proposed in this paper is useful to diagnose sensor faults of turbo-shaft engines. Finally, our work is summarized in the last section.

## 2. Turbo-Shaft Engine Model

The engine model accuracy directly determines the validity of the model-based method for sensor fault diagnosis. A dynamic coefficient approach is used to build up a simplified turbo-shaft on-board engine that satisfies the sensor fault diagnosis requirements. Taking into account of the structure and characteristics of turbo-shaft engine, the model is divided into a gas generator model and the power turbine model with loads.

### 2.1. Definition of Dynamic Parameters

A similar normalized gas generator speed *n_gc_* of turbo-shaft engine corresponds to a fuel flow *W_fc_* that is constant in the steady-state. The speed will change and the engine works in the dynamic operation when the actual fuel flow *W_fc_* is not match to the demand one. While *W_fc_* is bigger than the demand flue flow, the gas generator power is greater than the needs' power of the compressor and accessories, then gas generator speed increases.

The change of fuel flow Δ*W_fc_*, which influences the acceleration of *n_gc_*, is defined as follows:

(1) $$\begin{array}{c} W_{\text{fcs}}={\varphi_{1}}^{-1}(n_{gc})\\ \Delta W_{fc}=W_{fc}-W_{\text{fcs}} \end{array}$$

The dynamic parameter of gas generator speed at the time point *i*, denoted as *K_ng_*(*i*), is expressed:

(2) $$K_{n_{g}}(i)=\frac{n_{gc}(i+1)-n_{gc}(i)}{\Delta W_{fc}(i)\Delta t}$$

where Δ*t* is sampling time (in the paper Δ*t* = 0.12). Subscript *c* means that the parameter has been similarly normalized. Define the dynamic parameters of power turbine inner temperature *T*_45_*_c_* and power turbine output power *N_ec_* respectively as Equation (2), which are denoted as *K_T_*_45_(*i*) and *K_Ne_*(*i*):

(3) $$K_{T_{45}}(i)=\frac{T_{45c}(i)-T_{45cs}(i)}{\Delta W_{fc}(i)}$$

(4) $$K_{Ne}(i)=\frac{N_{ec}(i)-N_{\text{ecs}}(i)}{\Delta W_{fc}(i)}$$

where *T*_45_*_cs_* and *N_ecs_* are the steady state parameters of *T*_45_*_c_* and *N_ec_*, which can be interpolated by scheduling of *n_gc_* to *T_45c_*, *n_gc_* to *N_ec_*.

### 2.2. Simplified Gas Generator Model

A simplified gas generator model above idle is established via the steady interpolation table and dynamic parameters. The inputs of the model are *W_f_*, atmospheric temperature *T*_1_, and atmospheric pressure *P*_1_, and the outputs are *n_g_*, *T*_45_. The detailed program to calculate *n_g_* is as follows:

Step 1. Give the initialized parameters of gas generator model, such as *n_g_*(0), *T*_45_(0), *P*_3_(0), and similarly normalize the model parameters based on *T*_1_ and *P*_1_.
Step 2. Calculate the increase of fuel flow via Equation (2), and judge whether the operation conditions of the gas generator are in steady state or dynamic operation.
Step 3. Interpolate *K_ng_* by *n_gc_*, and compute the acceleration of gas turbine speed at this moment:
  (5) $${\dot{n}}_{gc}=K_{n_{g}}\Delta W_{fc}$$
Step 4. Calculate *n_gc_*(*i*+1) with integral of *ṅ_gc_*, and do a similar inverse transformation:
  (6) $$n_{gc}(i+1)=n_{gc}(i)+{\dot{n}}_{gc}\Delta t$$

Then return to step 1 to iterate.

The calculation method of *T*_45_ is similar to that of *n_g_*. Taking the current gas generator speed as an index, we search *K_T_*_45_ and *T*_45_*_cs_* from the interpolation tables and then calculate the *T*_45_*_c_* by the expression that follows:

(7) $$T_{45c}=T_{45cs}+K_{T_{45}}\Delta W_{fc}$$

### 2.3. Simplified Power Turbine Model with Loads

Combining the load characteristics with the rotor dynamics equation, the simplified power turbine model with loads is built up via the steady interpolation table and dynamic parameters. The inputs of the model are *n_gc_*, Δ*W_fc_*, *T*_1_, *P*_1_, and load angle *α*, the outputs are *Ne* and power turbine speed *n_p_*. The calculation procedure for *Ne* is the same as that of *T*_45_. The following equations solve for the parameter *Nec*:

(8) $$\text{Nec}=\text{Nec}s+K_{Ne}\cdot \Delta W_{fc}$$

The process of calculating *n_p_* contains two stages: one from the ground idle to flight idle, the other from flight idle to maximum state. In the first stage, dynamic operation of a power turbine model with loads is assumed with the load angle *α* and torque Δ*M* as constants, *α* = 0. The acceleration of *n_p_* satisfies the following equation:

(9) $$\Delta M=J_{Pt}\cdot {\dot{n}}_{p}$$

where *J_Pt_* is the rotation inertia of the power turbine shaft. *n_p_* can be acquired by the integral of *ṅ_p_*, and *n_p_*(0) is the initial value of *n_p_*:

(10) $$n_{p}=n_{p}(0)+\int_{0}^{t}{\dot{n}}_{p}dt$$

In the second stage, the state of the turbo-shaft engine is determined by the load angle. Power demands of power turbine shaft are changed with *α*, and can be interpolated from the load characteristics map:

(11) $$Nv=f(\alpha ,n_{p})$$

When the power turbine shaft power is not balanced, *n_p_* will change based on the following rotor dynamic equation:

(12) $$Ne-Nv=J_{Pt}\cdot n_{p}\cdot {\dot{n}}_{p}$$

**Figure 1.**
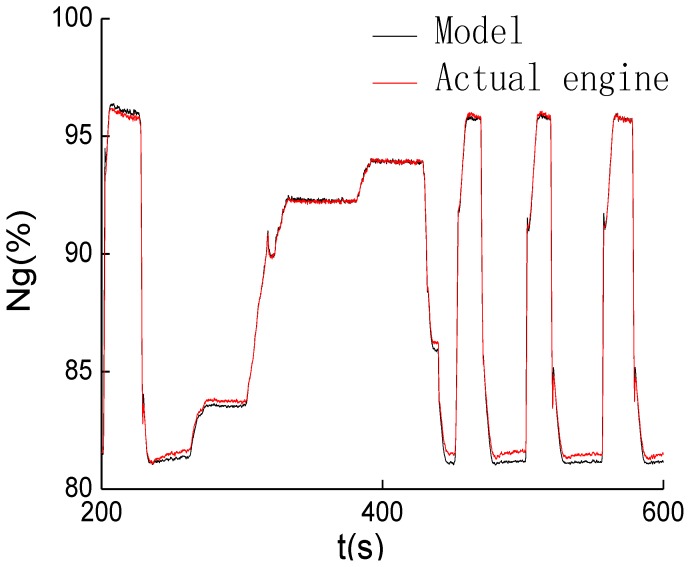
Outputs of *n_g_* by the model and turbo-shaft engine.

**Figure 2.**
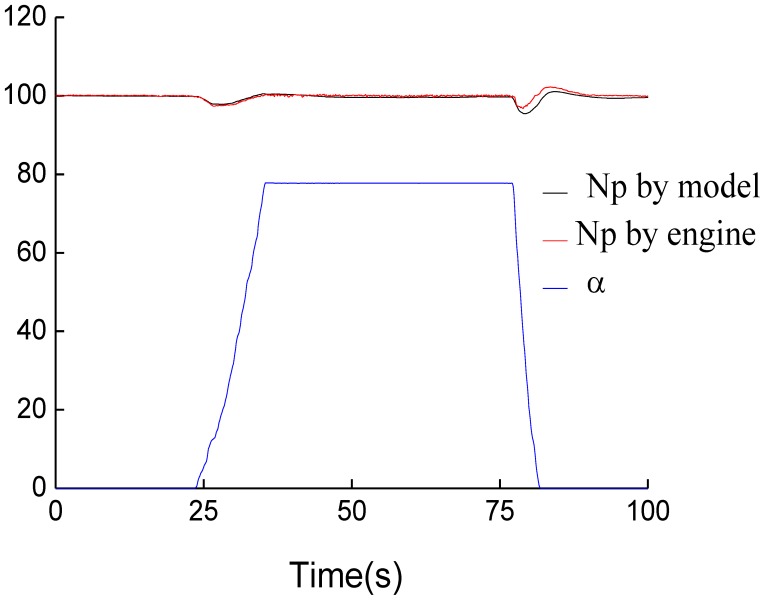
Outputs of *n_p_* by the model and turbo-shaft engine.

The term *n_p_* can be obtained from associated Equations (10–12). In order to evaluate the accuracy of simplified on-board turbo-shaft engine, experiments were carried out under the condition of *T*_1_ = 300.5 *K*, *P*_1_ = 102.4 kPa. Figure 1 represents the comparison *n_g_* of the model and actual turbo-shaft engine, and Figure 2 shows the comparison of *n_p_* of the model and turbo-shaft engine.

As seen from Figures 1 and 2, gas generator speed and power turbine speed calculated by model could track those of the actual engine. The relative steady errors of *n_g_* and *n_p_* between engine model and actual engine are no more than 0.5%, respectively, while the dynamic errors are less than 2%.

## 3. Sensor Fault Diagnosis Based on Simplified On-Board Model

**Figure 3.**
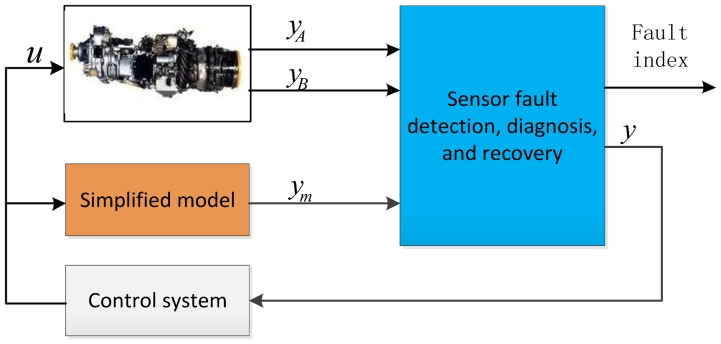
Sensor fault detection, diagnosis, and recovery principle.

The objective of on-board diagnosis for sensors is to detect and isolate faults as early as possible. Figure 3 represents the structure of the on-board diagnosis system composed of the simplified turbo-shaft engine and fault detection, diagnosis logic. It utilizes the simplified model as an analytical third channel for the turbo-shaft engine application. When the discrepancy among the triplex channels exceeds a tolerance level, the sensor fault diagnosis logic determines the cause of the difference.

### 3.1. Pretreatment for Measurements

The data acquired by sensors can't be directly used for engine health management or control, as the sensor measurements involve not only the useful signals but also measurement noise and other interference information, which will result in sensor fault diagnosis failures. Therefore, it is necessary to undertake a pretreatment process involving outlier elimination and smoothing of the original data.

**Figure 4.**
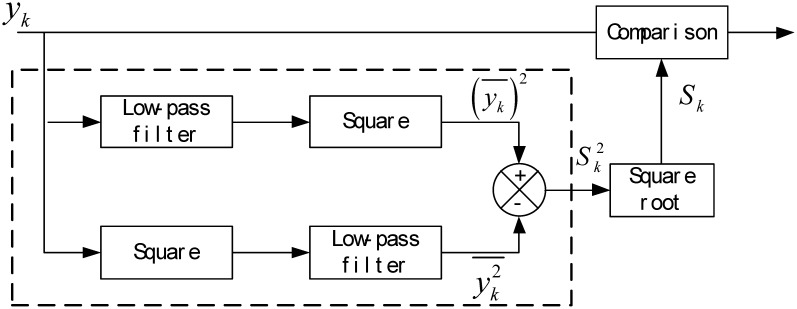
Framework of outlier discrimination and elimination.

For this we use a statistical method to discriminate and eliminate outliers, the framework of which is as shown in Figure 4. Two low-pass filters are introduced in the program, and their outputs are the smooth estimates of the measurements. The dashed box in the figure produces the updated value of the sample variance, which can be used to obtain the threshold for determining outlier at the next time. The detailed procedure of outlier discrimination and elimination is given out as follows.

Step 1. Smooth the sensor measurement by five near points at *k*:
  (13) $$\bar{y_{k}}=\frac{y_{k-2}+y_{k-1}+y_{k}+y_{k+1}+y_{k+2}}{5}$$
Step 2. Calculate the sums of squares of these five sensor measurement, and then acquire smooth value:
  (14) $$\bar{y_{k}^{2}}=\frac{y_{k-2}^{2}+y_{k-1}^{2}+y_{k}^{2}+y_{k+1}^{2}+y_{k+2}^{2}}{5}$$
Step 3. Define the variance $S_{k}^{2}$ as follows:
  (15) $$S_{k}^{2}={\left(\bar{y_{k}}\right)}^{2}-\bar{y_{k}^{2}}$$
Step 4. Check out the next sensor measurement, if it does not belong to the range $\left[{\bar{y}}_{k}-KS_{k},{\bar{y}}_{k}+KS_{k}\right]$, then is denoted as outlier, where *K* equals to 5.
Step 5. Eliminate the outlier and replace it with the following value:
  (16) $$y_{k+1}=\frac{y_{k-1}+y_{k}}{2}$$

**Figure 5.**
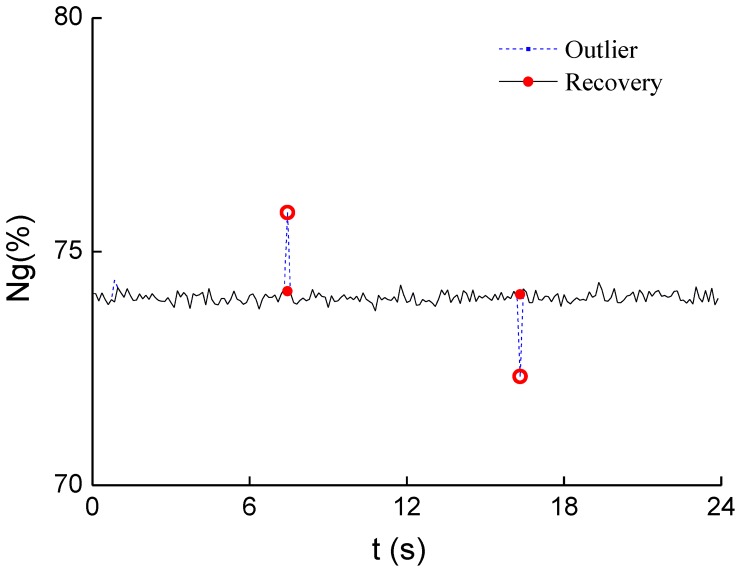
Simulation results of outlier discrimination and elimination.

Gas turbine speed is directly influenced to the power of turbo-shaft engine, hence, the experiment to validate the proposed method is focused on the parameter *n_g_*. Figure 5 shows the simulation of outlier discrimination and elimination for the parameter *n_g_*.

From the figure we can see that there are two outliers identified, and both of them are replaced with the new values within the rational range. Exponential smoothing is used to reduce the measurement noise in the paper.

**Figure 6.**
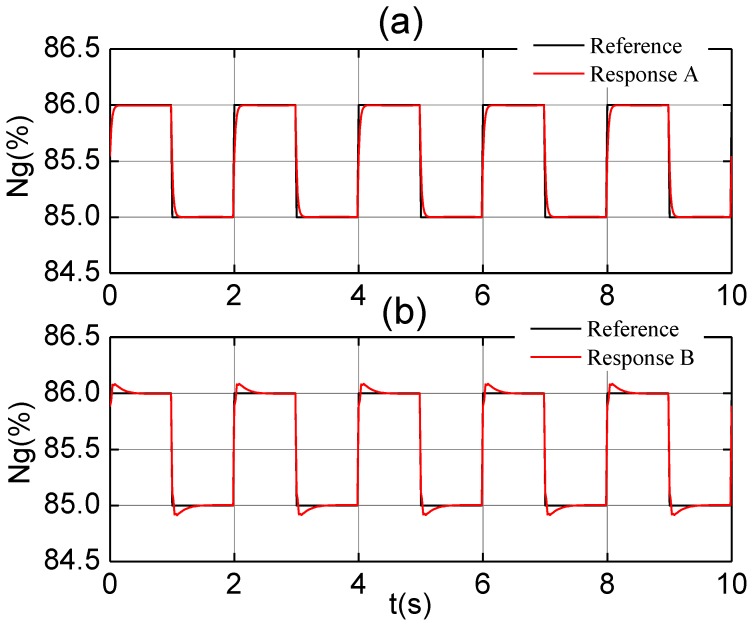
The response to step input by (**a**) PI controller with no delay; and (**b**) PID controller with delay.

The control system should maintain the parameter *n_g_* to the reference under the gas turbine mode, for which proportional plus integral (PI) controller is used. When proportional gain *K_p_* = 200 and integral gain *K_I_* = 600, the step response for a 1% step input under the steady state of *ng*% = 85% is shown in Figure 6(a). In order to discriminate and eliminate outliers, the sensed data *n_g_* is held for two time steps to determine the variances in Equations (14–16). If we still select PI controller, the control system will become unsteady. Therefore, proportional-integral-derivative (PID) controller is designed, we set *K_p_* = 80, *K_I_* = 400, and *K_D_* = 0.7. The overshoot of the 1% step response is less than 10%, the steady state is attained in 0.5 s, and the steady-state error is no more than 2%, as shown in Figure 6(b).

### 3.2. Sensor Fault Diagnosis Principles

The sensor fault detection and diagnosis logic compares the triplex channels (*y_A_*, *y_B_*, *y_m_*) and determines a root cause when an anomalous signature is detected in these channels. The duplicate channels (*y_A_*, *y_B_*) are the sensor measurements from the turbo-shaft engine, and *y_m_* is the output from the model. There are multiple comparisons that are carried out by the logic. The first comparison is a cross-check between channels A and B. There are a total of three engine parameters (*n_g_*, *T*_45_, *n_p_*) which are measured by three dual-channel sensors. For each measured parameter, the residual is defined as follows:

(17) $$r^{i}=\frac{\left|{y^{i}}_{A}-{y^{i}}_{B}\right|}{\sigma_{i}}$$

where *σ_i_* indicates the standard deviation of the *i^th^* sensor measurement uncertainty. The residual in Equation (17) is called the dual-channel residual. The dual channel residual for each sensor is compared against a corresponding threshold $\tau_{\mathrm{Dr}}^{i}$, If the dual channel residual does not exceed the threshold, the redundant measurements on both channels are acceptable. Otherwise, at least one of the dual channels is faulty. When a dual channel fails, the sensor measurement is replaced with the model output. The process can only determine whether at least one channel of the dual-channel sensor is faulty, but not which channel is faulty. Therefore, the other two sensor fault indicators are introduced, and the comparison of the dual channels against the model output is given out as follows:

(18) $$\begin{array}{c} {r^{i}}_{A}=\frac{\left|{y^{i}}_{A}-{y^{i}}_{m}\right|}{\sigma_{i}}\\ {r^{i}}_{B}=\frac{\left|{y^{i}}_{B}-{y^{i}}_{m}\right|}{\sigma_{i}} \end{array}$$

The residual in Equation (18) is called the analytical residual. The analytical residual computed for each channel of each sensor is compared against the threshold $\tau_{AR}^{i}$. The simplified model generates the expected output values of the engine operating without any faults. If an analytical residual exceeds a threshold, it indicates the existence of an anomaly.

**Figure 7.**
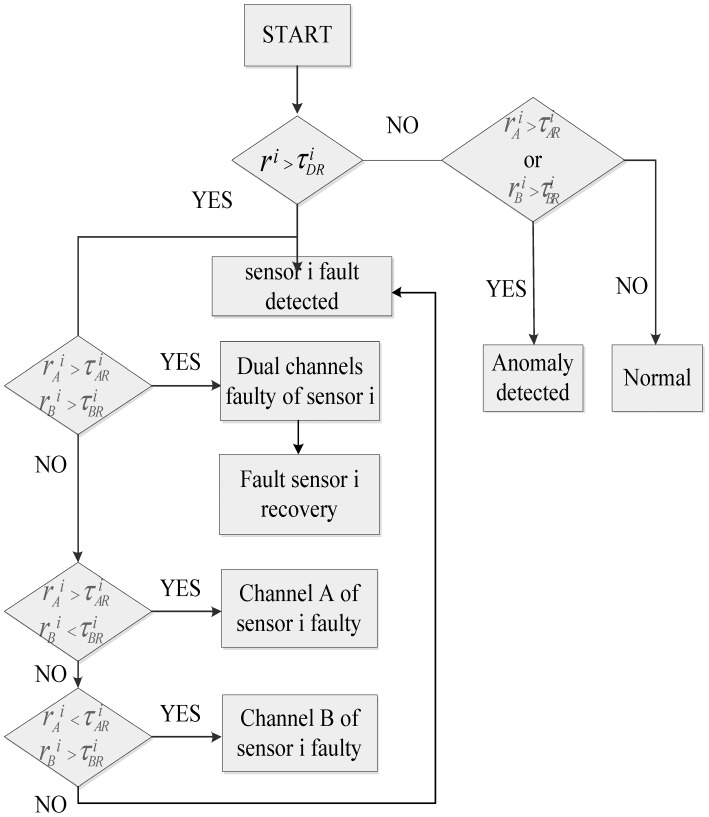
Dual-channel sensor fault detection, diagnosis, and recovery logic.

The sensor fault diagnosis logic can fulfill the following functions: (1) sensed data is detected; (2) sensor fault diagnosis; (3) faulty sensor recovery; or (4) anomaly detection. A flow chart of the sensor fault diagnosis logic is given in Figure 7.

The sensor fault detection, diagnosis, and recovery logic indicates that a sensor fault is detected when one of the dual-channel residuals in Equation (17) exceeds the threshold, but all analytical residuals in Equation (18) remain below the threshold. In this condition, the identity of a faulty dual channel sensor is determined, but the identity of its failed channel cannot be determined, and the faulty sensor cannot be isolated too.

The logic indicates that a sensor fault is isolated when the dual channel residual of a particular sensor exceeds the threshold, and also, this sensor's analytical residual exceeds the threshold in either one or both channels. If the threshold violation of the analytical residual occurs only in one channel, the channel that caused this violation is identified as the faulty one. If the threshold violation occurs in both channels, both channels of this particular sensor are considered faulty. Thus, the identity of a faulty sensor and the identity of its failed channel are determined. When both channels of sensor measurement are faulty, the faulty sensor signal used for control system is replaced by the model output. If the dual-channel residual of a particular sensor doesn't exceed the threshold, and this sensor's analytical residual exceeds the threshold in either one channel, some other type faults might happen, otherwise, turbo-shaft control system works normally.

### 3.3. Sensor Fault Threshold Selection

Sensor fault threshold selection directly affects the results of sensor fault diagnosis. In order to make the system able to detect the sensor fault with little amplitude changes, the threshold range needs to be set more smaller, while it will result in misdiagnosis. Therefore, a rational threshold selection is necessary for improving the accuracy of sensor fault diagnosis. In this paper, the threshold is selected by the statistical characteristics of sensor measurement noise and the model errors.

The engine outputs measured by sensors with dual-channels A and B are expressed as follows:

(19) $$\begin{array}{c} y_{A}=y_{m}+\Delta y+v_{1}\\ y_{B}=y_{m}+\Delta y+v_{2} \end{array}$$

The parameters *y_m_* and Δ*y* separately represent the turbo-shaft engine model output and the modeling errors. The parameters *v*_1_ and *v*_2_ are the zero-mean, normally distributed white noise that corrupts the measurements on dual-channels, and independent each other, denoted as *v*_1_∼*N*(0,*σ*^2^), *v*_2_∼*N*(0,*σ*^2^). The probability density function of dual-channel random residual Δ*v* = *v*_1_ – *v*_2_ is as:

(20) $$f\left(\Delta v\right)=\int_{-\infty }^{+\infty }f(v_{1})\cdot f(v_{1}-\Delta v)dv_{1}=\frac{1}{2\sqrt{\pi }\sigma }e^{-\frac{\Delta v^{2}}{4\sigma^{2}}}$$

The function *f* is the probability density function of the parameters *v*_1_ and *v*_2_. From the Equation (20), we can see Δ*v* = *σ*∼*N*(0,2). Then the probability density function and the distribution function of dual-channel residual *r* are respectively in the following form:

(21) $$\begin{array}{cc} f(r)=\frac{1}{\sqrt{\pi }}e^{-\frac{r^{2}}{4}} & \left(0<r<+\infty \right) \end{array}$$

(22) $$F(r)=\Phi (\frac{r}{\sqrt{2}})-\Phi (-\frac{r}{\sqrt{2}})$$

The function Φ is the distributing function of standard normal distribution function, In order to make sure as less misdiagnosis rate as possible, the following equation $F(3\sqrt{2})=99.7\%$ is satisfied, and *τ_DR_* = 4.5 in the article.

The threshold of the analytic residual is determined not only by the measurement noise but also by the modeling errors. The analytic residual can be expressed in the following form:

(23) $$\begin{array}{c} r_{A}=\left|\frac{v_{1}+\Delta y}{\sigma }\right|\\ r_{B}=\left|\frac{v_{2}+\Delta y}{\sigma }\right| \end{array}$$

Considering the random variables *v*_1_/*σ*, *v*_2_/*σ* follow the standard normal distribution, and 3*σ* criterion, the threshold *τ_AR_* and *τ_BR_* can be calculated via following equations:

(24) $$\begin{array}{c} \tau_{AR}=\left|\frac{\Delta y}{\sigma }\right|+3\\ \tau_{BR}=\left|\frac{\Delta y}{\sigma }\right|+3 \end{array}$$

From Section 2, we have obtained the modeling errors under steady state and dynamic state. Then both of dual-channel threshold and analytic threshold can be computed. Tables 1 and 2 show statistical results of sensor measurement noise under four operation conditions, and the corresponding thresholds.

## 4. Experiments on a Turbo-Shaft Engine

The capability of sensor the FDD system based on a simplified on-board model to detect, diagnose and recovery a biased sensor is evaluated. A bias is injected into channel A or B of a single sensor *n_g_* at a time, and the health condition of the engine is set to the nominal health. There are two types of sensor faults to be simulated: the step fault, and the drift fault. In the presence of a sensor bias, the closed-loop system is trimmed at a cruise operating condition. When any of the signals (dual-channel residuals and analytical residuals) exceed a threshold for 20 consecutive time steps (sampling time 0.12 s), a threshold violation is declared. Based on the threshold violations occurring from the engine, the fault diagnosis logic gives out the sensor fault diagnosis result.

**Figure 8.**
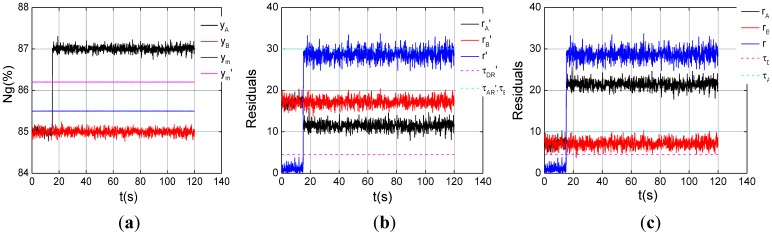
One channel sensor *n_g_* step fault under the steady state of *ng*% = 85%. (**a**) Triplex channel outputs; (**b**) Step fault indication by SPSO-SVR; (**c**) Step fault indication by simplified model.

Experiments on one channel with a step fault and a drift fault under the steady-state of *ng*% = 85% are carried out. The magnitude of 2% step fault was injected into channel A at 15s in Figure 8(a). The dual-channel residual *r* grows about 28.7, and the analytic residual *r_A_* about 21.2, bigger than their thresholds *τ_DR_* and *τ_AR_* at 15 s, as shown in Figure 8(c). However the analytic residual *r_B_* does not violate its threshold *τ_BR_*. Therefore channel A fault can be determined by the logic, and the diagnostic root cause is consistent with the sensor fault set. The SPSO-SVR method for sensor fault diagnosis in [28] is used for comparison, and the input and output of SPSO-SVR estimator are [*Wf*(*k*−1), *Wf*(*k*)] and *ng*(*k*), respectively. The SPSO-SVR steady estimation error is more than the simplified model one, so does the analytic residual threshold. The dual-channel residual *r*′ grows about 28.7, the analytic residual $r_{A}^{'}$ decreases to 12 at 15 s, and the analytic residual $r_{B}^{'}$ keeps 17, as shown in Figure 8(b). There are no residuals exceed themselves' thresholds and no sensor faults are determined, but it is not consistent with the truth and the misdiagnosis is produced.

**Figure 9.**
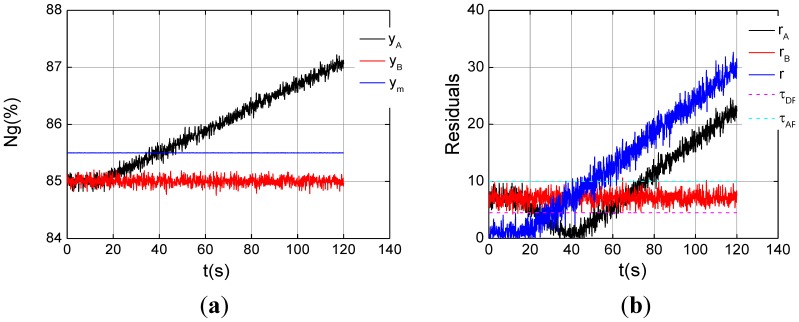
One channel sensor *n_g_* drift fault under the steady state of *ng*% = 85%. (**a**) Triplex channel outputs; (**b**) Drift fault indication.

In Figure 9(a), the magnitude 0.02%/s drift fault is introduced into channel A from 15 s. The dual-channel residual *r* exceeds the *τ_DR_* about at 30 s, and one of the analytic residual, *r_A_*, violates its threshold *τ_AR_* at 75 s, as shown in Figure 9(b). Channel A fault is recognized about 2 s after 75 s.

**Figure 10.**
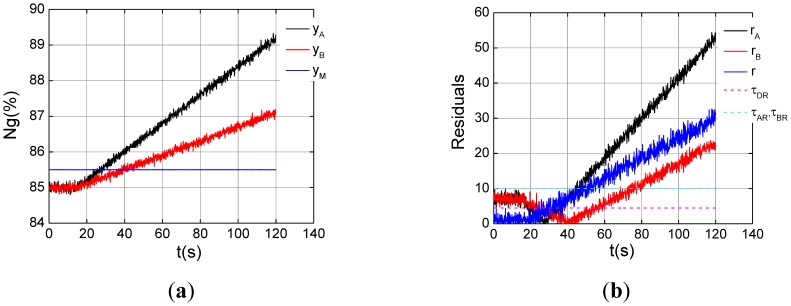
Dual channel fault with different drift velocities under the steady state of *ng*% = 85%. (**a**) Triplex channel outputs; (**b**) Drift fault indication.

**Figure 11.**
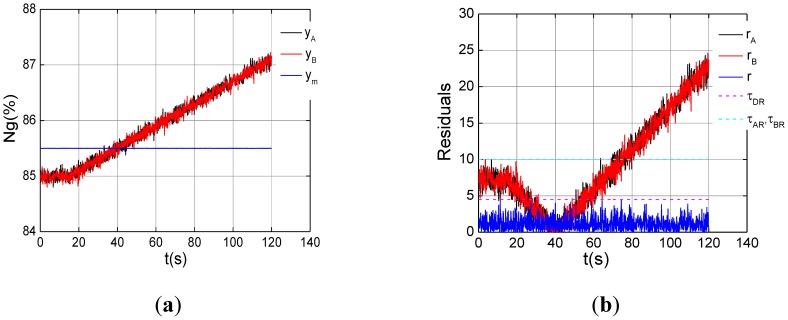
Dual channel fault with the same drift velocities under the steady state of *ng*% = 85%. (**a**) Triplex channel outputs; (**b**) Drift fault indication.

Two experiments on dual channel with drift faults under the steady-state of *ng*% = 85% are carried out. The faults with different drift velocities are introduced into dual channels in Figure 10(a), channel A is 0.04%/s, and channel B 0.02%/s. As can be seen from Figure 10(b), three fault indicators exceed their thresholds about at 80s, dual channel faults is determined 2.4 seconds later via the logic. Both of the channels are isolated, and the sensor signal will be replaced with the model output. In Figure 11(a), we can see that the same drift faults occur in the dual channel, and both analytic residuals violate their threshold while the dual channel residual is still below its threshold. An anomaly is detected, but which sensor is fault can't be recognized by the fault indicators in Figure 11(b).

**Figure 12.**
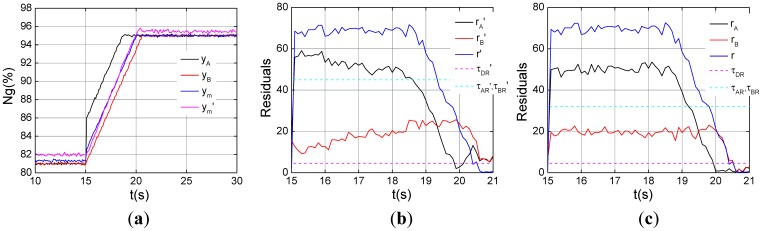
Channel A with the step fault in the dynamic operation. (**a**) Triplex channel outputs; (**b**) Step fault indication by SPSO-SVR; (**c**) Step fault indication by simplified model.

Sensor fault diagnosis logic for turbo-shaft engines is validated under the steady state from the above experiments. Modeling errors of dynamic operation are much more numerous than those of steady state. In order to evaluate the ability of sensor fault diagnosis logic under dynamic operation, the following experiment is designed. When the gas generator speed increases from 81% to 95%, the step fault is injected into the channel A in Figure 12(a).

Sensor misdiagnosis will happen under the steady state of *ng*% = 81% by SPSO-SVR, which can be obtained the same process as Figure 8(b). The SPSO-SVR dual-channel residual *r*′ grows about 70, analytic residual *r_A_*′ about 50, bigger than their thresholds Δ *τ_DR_*′ and *τ_AR_*′ at 15 s. However the analytic residual *r_B_*′ does not violate its threshold *τ_BR_*′, as shown in Figure 12(b). Therefore channel A fails according to the logic, and the diagnostic root cause is consistent with the sensor fault set. As can be seen from the Figure 12(c), the same determination can be obtained.

## 5. Conclusions

The sensor FDD system based on a simplified on-board model described in this paper is proposed and developed to diagnose turbo-shaft engine faults on-line. A simplified on-board model for turbo-shaft is designed, and it contains two segments: gas generator model and power turbine model with loads. The sensor fault diagnosis system utilizes dual-channel sensor measurements and also the output of a simplified on-board engine model as the analytical third channel. Through the comparison of triplex channels, the system diagnoses two types of faults in sensors.

The sensor fault detection, diagnosis, and recovery logic is designed, and the sensor FDD system is evaluated extensively at a cruise operating condition using simulated fault cases. Compared to the SPSO-SVR method, the proposed one exhibited its capability to identify a faulty dual-channel sensor and its failed channel at a reasonable fault level. The sensor FDD system based on a simplified on-board model can be used under the steady state and dynamic operation. The diagnostic capability of the sensor fault diagnosis system establishes a benchmark for on-line diagnostics. Any improvement made through the application of advanced diagnostic techniques can be evaluated against this benchmark.

## Figures and Tables

**Table 1.** Statistical results of sensor *n_g_* for the turbo-shaft engine.

**State**	**Standard deviation σ**	**Maximum bias**	***τ****_DR_*	***τ****_AR_*, ***τ****_BR_*
Flight idle	0.045	0.15	4.5	14
85% of *n_g_*	0.070	0.25	4.5	10
99.6% of *n_g_*	0.082	0.25	4.5	9
Dynamic operation	0.069	0.23	4.5	32

**Table 2.** Statistical results of sensor *T*_45_ for the turbo-shaft engine.

**State**	**Standard Deviation σ**	**Maximum Bias**	***τ****_DR_*	***τ****_AR_*, ***τ****_BR_*
Flight idle	0.085	0.27	4.5	9
89.0% of *n_g_*	0.113	0.32	4.5	8
100.1% of *n_g_*	0.095	0.33	4.5	8
Dynamic operation	0.114	0.32	4.5	21
